# Purple Urine Bag Syndrome in End-Stage Renal Disease: Recognizing the Unexpected

**DOI:** 10.7759/cureus.85178

**Published:** 2025-06-01

**Authors:** Pallavi Shirsat, Kunal Sonavane, Bhawna Agarwal, Gautam Agrawal, Harikrishna Choudary Ponnam, Laxmi Sakamuri

**Affiliations:** 1 Nephrology, Minden Medical Center, Minden, USA; 2 Internal Medicine, Willis Knighton Medical Center, Bossier City, USA; 3 Internal Medicine, University of Pittsburgh Medical Center McKeesport Hospital, McKeesport, USA; 4 Nephrology, Independence Health System, Greensburg, USA; 5 Internal Medicine, Summa Health, Akron, USA

**Keywords:** maintenance hemodialysis, neurogenic bladder dysfunction, pubs, purple urine bag, urinary tract infection

## Abstract

Purple urine bag syndrome (PUBS) is an uncommon but striking phenomenon seen primarily in elderly patients with neurogenic bladder and long-term indwelling Foley catheters. In some cases, it may be the only clinical manifestation of a urinary tract infection (UTI). The characteristic purple discoloration results from metabolic byproducts such as indigo and indirubin, formed through bacterial enzymatic activity in the urine. Though benign in itself, the condition can cause significant anxiety in patients, caregivers, and healthcare providers unfamiliar with it. We present the case of an elderly female dialysis patient with a neurogenic bladder and chronic Foley catheter use, whose only sign of recurrent UTIs was the development of purple urine. Notably, there is only one other reported case of PUBS in a patient undergoing dialysis, highlighting the rarity of this presentation. Through this report, we aim to underscore the importance of recognizing PUBS as a potential early indicator of infection, in order to facilitate prompt management and prevent unnecessary physical and psychological stress.

## Introduction

Purple urine bag syndrome (PUBS) is a rare clinical condition first described in 1978 [1,2]. It is typically asymptomatic, and its prevalence can reach up to 9.8% among institutionalized patients with long-term indwelling urinary catheters [1]. It is most commonly observed in elderly, debilitated individuals with a neurogenic bladder requiring prolonged Foley catheterization. PUBS is frequently associated with urinary tract infections (UTI) and may, in some cases, be the sole presenting sign [3]. The unusual discoloration often causes unnecessary alarm among patients and caregivers if not properly recognized. We describe the case of a 70-year-old female on chronic hemodialysis who developed PUBS as the only manifestation of her UTI.

## Case presentation

**Figure 1:**
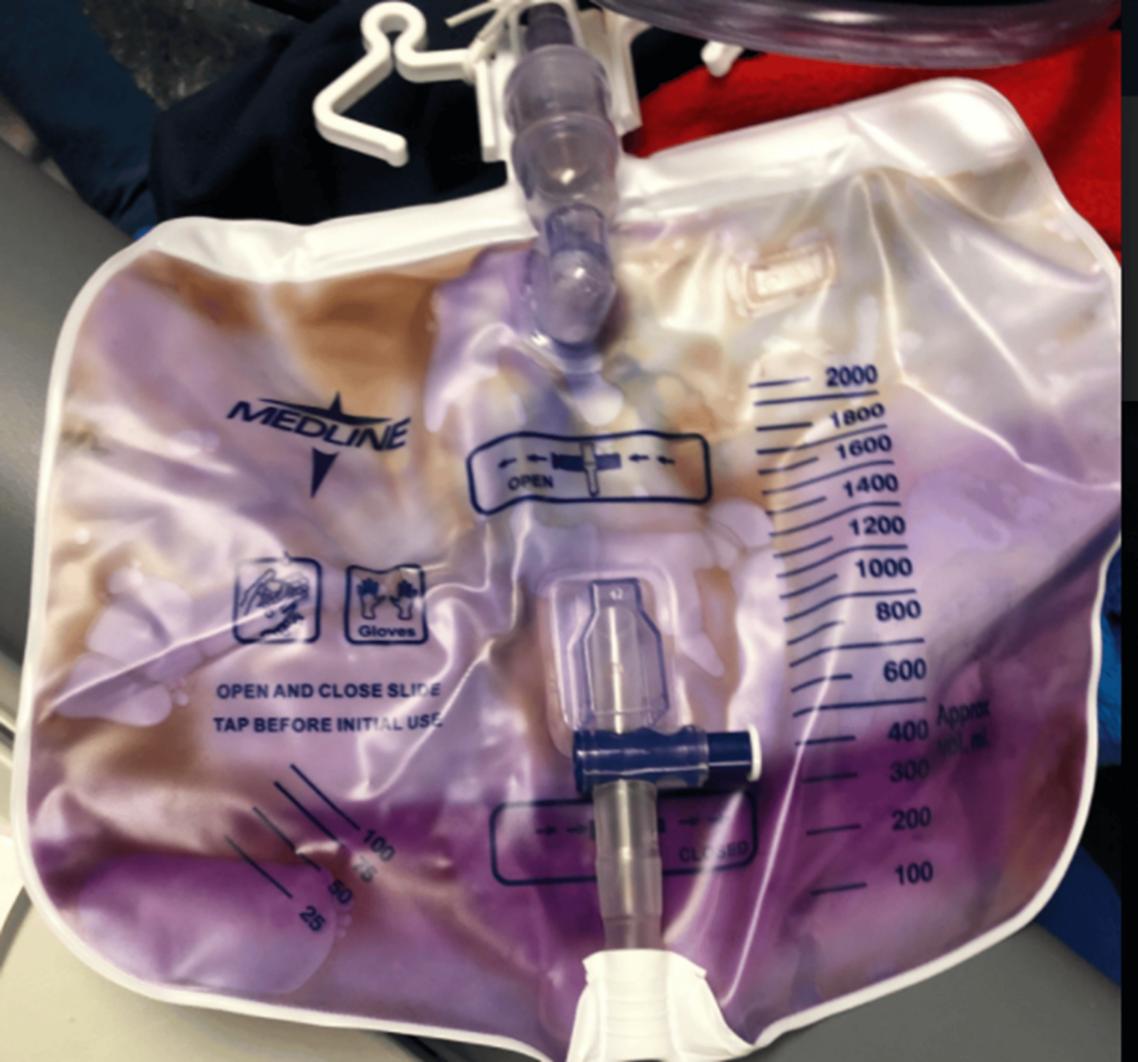
Purple discoloration of urine noted in the Foley catheter bag of the patient This represented her first documented episode of purple urine bag syndrome (PUBS), which caused considerable distress. Following replacement of the catheter, tubing, and collection bag, and initiation of antibiotic therapy, the discoloration resolved completely

The patient was a 70-year-old female with end-stage renal disease (ESRD) secondary to diabetic nephropathy, receiving in-center hemodialysis. She had a history of neurogenic bladder managed with a chronic indwelling Foley catheter. The patient presented to the dialysis unit after noticing purple discoloration in her urine collection bag (Figure 1), which caused her significant distress. She reported associated mild lower abdominal discomfort and fatigue for the preceding two days, as well as recent constipation (her last bowel movement had been two days ago). She denied experiencing fever, chills, nausea, vomiting, or changes in appetite. Given the recent onset of her symptoms and her documented compliance with the dialysis regimen, uremia was excluded as a contributing etiology.

Her past medical history included hypertension, coronary artery disease, and an abdominal aortic aneurysm under surveillance. Home medications included aspirin 81 mg daily, carvedilol 25 mg twice daily, atorvastatin 40 mg nightly, nifedipine ER 60 mg twice daily, and sertraline 25 mg daily. She denied taking any other supplements or herbs. Dialysis-administered medications included cinacalcet 30 mg three times weekly, calcitriol 0.75 mcg three times weekly, and IV iron (Venofer) and erythropoiesis-stimulating agents (Mircera) as per the dialysis unit protocol.

On examination, she was afebrile (98.4°F), with blood pressure of 110/66 mmHg, heart rate of 73 bpm, and respiratory rate of 16. She appeared well-nourished and in no acute distress. Physical exam was notable for conjunctival pallor and mild suprapubic tenderness on deep palpation, without rebound or guarding. Bowel sounds were present; the remainder of the examination was unremarkable.

Laboratory evaluation revealed a WBC count of 8,070/μL, hemoglobin of 10.4 g/dL, hematocrit of 32.2%, and platelets of 228,000/μL. She did not exhibit leukocytosis. Urinalysis demonstrated alkaline urine (pH 8.5), positive leukocyte esterase, and positive nitrites (Table 1). Urine culture grew *Proteus mirabilis* and *Klebsiella oxytoca*. Blood cultures and imaging were deferred, as the patient exhibited no additional signs of sepsis, such as high-grade fever or hemodynamic instability, making the likelihood of systemic infection low.

**Table 1:** Urine analysis findings

Laboratory test	Patient value	Reference range
Color	Dark yellow	Yellow
Appearance	Turbid	Clear
Specific gravity	1.015	1.003-1.030
pH	8.5	5.0-9.0
Leukocyte esterase	3+	Negative
Nitrite	Positive	Negative
Protein	2+	Negative
Glucose	Negative	Negative
Ketones	Trace	Negative
Urobilinogen	1	0.2-1.0 EU/dL
Bilirubin	1+	Negative
Blood	Negative	Negative

Given its ease of administration during dialysis sessions, gentamicin was empirically selected and administered at a dose of 160 mg intravenously (IV) once, followed by 80 mg IV three times weekly post-dialysis for one week. Traditional dosing with a loading dose of 2 mg/kg followed by 1 mg/kg post-dialysis was chosen, as it is been associated with a comparatively lower risk of nephrotoxicity than the extended-interval dosing regimens. Her Foley catheter and urine collection system were replaced, and both her symptoms and urine discoloration resolved within 48 hours of initiating treatment.

Her Foley catheter and collection system were routinely changed once a month by home health services, in accordance with their standard protocol. She continued to typically experience episodes of PUBS approximately three to four times per year, each of which was managed with culture-guided antibiotic therapy and replacement of the catheter and collection system, until she eventually became anuric and the episodes ceased.

## Discussion

PUBS is an unusual but important indicator of UTI in elderly catheterized patients, particularly women with neurogenic bladder [3]. Studies suggest that its incidence may be as high as 10% among institutionalized patients with long-term indwelling urinary catheters [2]. The condition results from the bacterial metabolism of tryptophan. In the gut, tryptophan is converted into indole, which is absorbed, metabolized in the liver to indoxyl sulfate, and excreted in the urine. Certain bacteria, particularly *Proteus, Klebsiella, Providencia, and Morganella*, produce indoxyl sulfatase/phosphatase enzymes that convert indoxyl sulfate into indigo (blue) and indirubin (red) pigments, resulting in a purple hue [4].

Risk factors for PUBS include advanced age, female gender, chronic constipation, alkaline urine, poor catheter hygiene, and chronic kidney disease [5]. Among these, alkaline urine plays a critical role by facilitating the enzymatic conversion of indoxyl sulfate into indigo and indirubin pigments, which impart the characteristic purple discoloration to the urine [5]. Although PUBS itself is generally benign, it can be indicative of a significant underlying UTI and, in some cases, may be the sole presenting sign of infection [3]. If left unrecognized or untreated, PUBS can occasionally progress to severe complications such as urosepsis, which carries serious morbidity risks, particularly in vulnerable patient populations such as elderly individuals with multiple comorbidities and those requiring dialysis.

Therefore, maintaining a high index of clinical suspicion and conducting appropriate microbiological investigations are essential to guide targeted treatment and prevent potentially life-threatening complications [6]. Additionally, judicious use of antibiotics guided by culture results is critical to uphold antibiotic stewardship principles, minimize the development of resistance, and ensure optimal patient outcomes. Notably, our patient did not exhibit additional risk factors commonly associated with more complicated or drug-resistant PUBS, such as prolonged antibiotic use, underscoring the importance of individualized assessment in management. 

**Figure 2:**
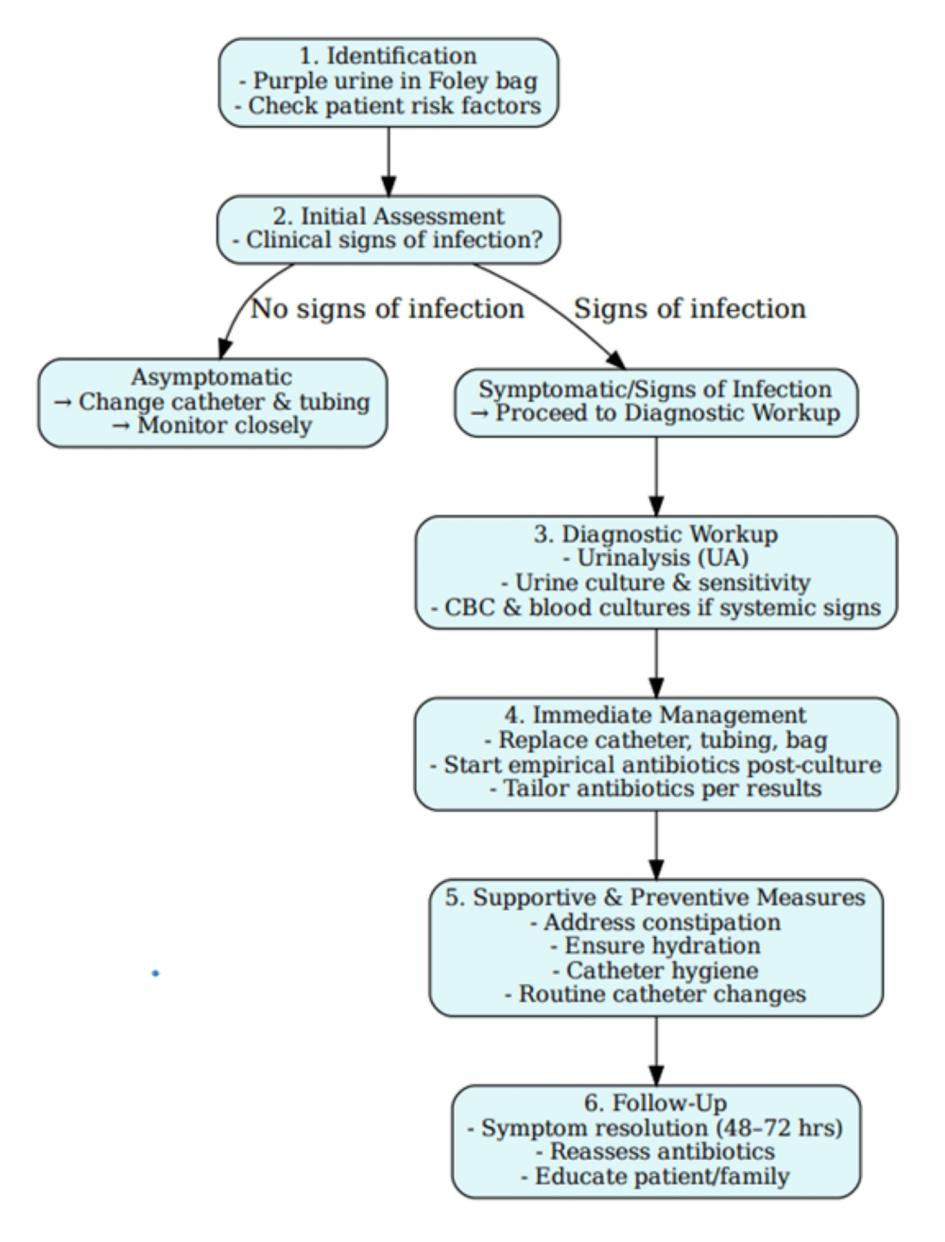
Clinical algorithm for the identification and management of purple urine bag syndrome (PUBS)

Figure 2 depicts a clinical algorithm outlining a step-wise approach towards the management of PUBS.

Other unusual catheter- or pigment-related syndromes have also been described, such as the “purple diaper syndrome” in infants and “blue diaper syndrome” [7], which result from metabolic or pigmentary abnormalities affecting urinary coloration. In nephrology, pigment-related findings may also include hematuria or myoglobinuria, highlighting the diverse clinical presentations of pigment alterations in urine.

There have been a few reported cases of PUBS in the literature [8,9]; however, awareness of this condition remains limited among clinicians and other healthcare providers. Through this report, we aim to highlight the benign nature of this dramatic presentation when diagnosed early and managed promptly.

## Conclusions

Clinicians should recognize that purple discoloration of urine in a catheter bag, particularly in elderly women with neurogenic bladder and long-term Foley catheter use, may signify an underlying UTI. Although striking in appearance, this condition is benign when promptly identified and appropriately managed with targeted antibiotic therapy and catheter system replacement. Preventive strategies - including maintaining proper catheter hygiene, effective management of constipation, and adherence to routine catheter care protocols - are based primarily on expert consensus and best clinical practices, as formal clinical guidelines specific to PUBS are limited. Despite multiple case reports, PUBS remains underrecognized, highlighting the need for increased awareness among both healthcare providers and patients. Further research and development of guidelines are warranted to standardize management approaches in these patients.
